# Supplementary material for: Factors associated with pneumococcal carriage and density in infants and young children in Laos PDR
Source: PLoS One. 2019 Oct 29;14(10):e0224392. doi: 10.1371/journal.pone.0224392 (PMC6818791; doi:10.1371/journal.pone.0224392)
Supplement: Attachment — Submitted filename: response to reviewers.docx [file pone.0224392.s002.docx]

**Response to reviewers**

*The authors thank the editor and reviewers for their consideration of our revised manuscript and the feedback provided. Please find a response to reviewer comments below. Line numbers refer to the clean, final version of the manuscript.*

Review Comments to the Author

Reviewer #1: The authors have addressed all my concerns, but I still have a minor suggestion. The range of each data of bacterial density can be provided to make the presenting data more clear.

*Authors’ response:*

*As suggested, we have added ranges of bacterial density data in the results text (lines 180 – 181) and to Table 5.*
